# Impact of reduced scan extent on tumor detection in ^68^Ga-PSMA PET/CT for biochemical recurrence of prostate cancer

**DOI:** 10.1186/s12880-026-02940-w

**Published:** 2026-10-21

**Authors:** Falk Gühne, Alexander Küppers, Christian Kühnel, Philipp Seifert, Martin Freesmeyer

**Affiliations:** University Hospital Jena, Clinic of Nuclear Medicine, Am Klinikum 1, 07747 Jena, Germany

**Keywords:** PSMA-PET/CT, Prostate cancer, Biochemical recurrence, Scan protocols, Efficiency

## Abstract

**Background:**

PSMA PET/CT plays an important role in biochemically recurrent prostate cancer (BCR-PCa). In general, PET/CT is performed as a limited or true whole-body examination. The majority of PET/CT scanners operate by measuring several bed positions or continuous motion with a limited axial field of view (FOV). Shortening the scan area thus results in a time saving, with unknown information loss regarding the detection of tumor manifestations.

**Methodology:**

This retrospective single-center study included 200 patients with BCR-PCa after initial curative therapy who underwent a ^68^Ga-PSMA-PET/CT scan from vertex to toe (true whole-body). Using retrospective subdivision into hypothetical partial body measurement protocols, the undetected tumor manifestations were examined. Classic PET/CT protocols were used: vertex to mid-thigh, skull base to mid-thigh, as well as CT-equivalent examination areas: chest/abdomen/pelvis (CAP), abdomen/pelvis (AP) and pelvis.

**Results:**

Of 200 patients with a median PSA of 1.1 ng/mL, 128 (64%) showed at least one structural correlate of BCR-PCa in the true whole-body scan. The overall detection rate did not change for limited classic PET-protocols, or CAP measurements. CAP scans would not have detected all tumor manifestations in 16 patients (8%) and not all tumor-affected organ systems in six patients (3%), AP scans in 41 patients (21%) and 18 patients (9%), pelvic scans in 61 patients (31%) and 37 patients (19%), respectively. PSA may contribute to the prospective restriction of the scan area. Patients with PSA ≤ 0.75 ng/mL showed no diagnostic gain from whole-body imaging. This means that in 45% of patients in our cohort, a limitation to CAP scan would have been possible without losing information.

**Conclusions:**

For a resource-saving use of routine PSMA-PET/CT, a scan from the vertex to the mid-thigh seems sufficient for detecting relevant metastases. For patients with low PSA levels, a more focused protocol that includes only the CAP region can be considered.

## Introduction

Prostate cancer (PCa) is one of the most common tumors and cancer related causes of death in the world with rising incidence [1]. Disease recurrence after initially curative resection or radiation occurs frequently, and is most commonly detected by rising prostate-specific antigen (PSA) levels during routine follow-up, referred to as biochemical recurrence (BCR) [2]. Identifying the structural correlate of BCR is essential for therapeutic decision-making, particularly when curative or metastasis-directed treatment remains feasible [3].

Positron emission tomography/computed tomography (PET/CT) has become an integral component of oncologic imaging. In particular, prostate specific membrane antigen (PSMA) ligand PET/CT is strongly recommended by current guidelines for patients with BCR-PCa due to its superior sensitivity [4]. In analogy to other PET/CT applications, PSMA-PET/CT is commonly performed as a whole-body examination. This contrasts with conventional radiological imaging modalities such as CT or MRI, which are typically tailored to specific anatomical regions based on the clinical question. Considering metastatic mechanisms and the characteristic pathways of PCa tumor spread, which usually involve the prostate bed, locoregional lymph nodes and the axial skeleton, while distal metastases – especially in the lower extremities – are rare, the necessity of extended whole-body imaging warrants reconsideration [5–7].

To date, protocol adaptations in PET/CT have largely been restricted to variations between true whole-body imaging from vertex to toe and limited whole-body imaging from skull base to mid-thigh [8, 9]. Although the scans from vertex to mid-thigh are generally considered sufficient in PSMA-PET/CT, the longitudinal scan extent remains substantial [10]. Further restriction of the scan range may improve efficiency but raises concerns about missing clinically relevant metastases.

Therefore, this study investigates the diagnostic impact of shortened PSMA-PET/CT protocols in patients with BCR-PCa. Specifically, we evaluate whether reduced scan extents affect detection of one structural correlate of BCR, overall number of tumor lesions, or involvement of additional organ systems, and whether scan protocols could be prospectively individualized with considerations of patient safety, cost, and resource efficiency.

## Methods

### Patients and inclusion

This retrospective single-center study evaluates PSMA-PET/CT scans performed in clinical indication; patients were not exposed to study specific radiation or examinations. The study was registered and proved by the local Ethics committee of the Friedrich-Schiller-University Jena under the registration number 2021-2209-Daten. A total of 1022 PSMA-ligand PET/CT examinations of 811 patients from the years 2013 to 2020 were screened. Data was included if: histologically confirmed prostate cancer; initially curative treatment; raised or persistent PSA levels; so far unknown distant metastases; true whole-body scan from vertex to toe. Follow-up scans of the same patient were excluded from the evaluation. No minimum threshold for the increase in PSA was defined; the examinations were conducted in a clinical setting, taking into account the information provided by the referring physician. An internal confirmation of the PSA level was not a prerequisite for the indication.

### PET/CT and data acquisition

All PET/CT examinations were interpreted by two nuclear medicine specialists with several years of experience in the use of PSMA-PET/CT. Focal PSMA-ligand uptake with or without morphological pathology within CT scan was rated as a tumor lesion if clinically plausible. PSMA-negative findings were not assessed. The images were reevaluated specifically for the study analysis. However, the investigators were not blinded to the initial clinical reports and PSA values. The investigators did not know the clinical outcomes or confirmation of actual tumor lesions. Consensus was obtained for every debatable case. Additional PET or contrast-enhanced CT scans were not considered. Patients’ characteristics were collected regarding disease and prior treatment as well as parameters of PET/CT acquisition. All PET/CT examinations were performed at the same scanner, a Biograph mCT 40 (Siemens Healthineers, Erlangen, Germany), in accordance to an internal standard operation procedure with a standardized activity of the PSMA-ligand and an uptake time of at least 60 min. Using a step-and-shoot PET system every bed position of 21.6 cm was scanned for 2 min with an overlap of 43% at each side. Due to changing availability, three different PSMA-ligands were used over the time, PSMA-617, PSMA-HBED/-11 and PSMA-I&T. All were radiolabeled with Gallium-68 (^68^Ga). PET scans were reconstructed iteratively (TrueX, Siemens Healthineers). CT was conducted as an auxiliary CT for attenuation correction. Additionally performed contrast-enhanced CT scans or PET scans of single body regions were not analyzed for this study. Every patient received 20 mg of furosemide i.v. to promote urinary clearance of the radiotracer. The arms were positioned alongside the torso for the scanning procedure and were completely included. The true whole-body scans were retrospectively partitioned in anatomically oriented parts (Table 1) for the allocation of the tumor lesions.

**Table 1:** Definition of anatomical limits of body parts

	Cranial boundary	Caudal boundary
Head/neck	Vertex of the skull	Apex of the lung
Thorax	Apex of the lung	Phrenicocostal recess
Abdomen	Dome of the diaphragm	Iliac crest
Pelvis	Iliac crest	Inferior ramus of the pubic bone
Thighs	Inferior ramus of the pubic bone	Knee joint
Lower leg	Knee joint space	Sole of the foot

**Fig. 1:**
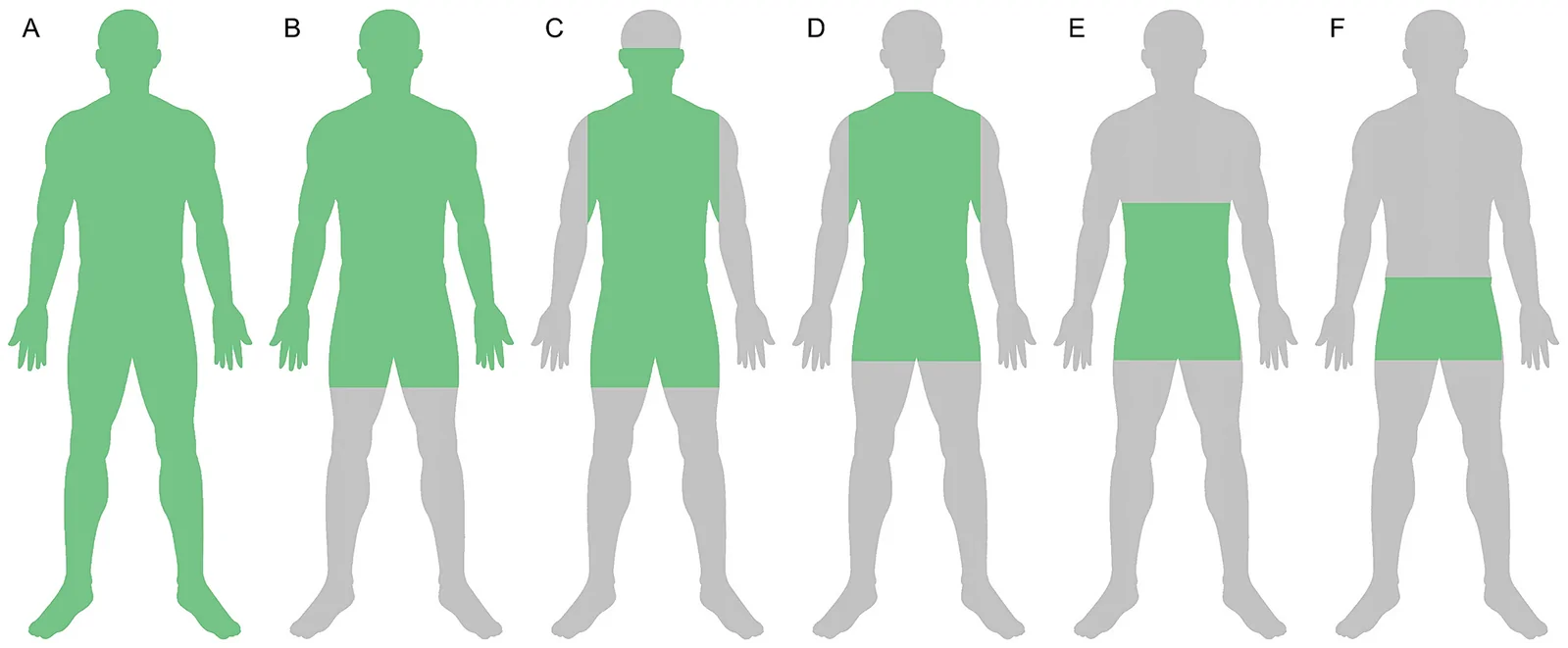
Depiction of the interpreted body region per protocol colored in green and excluded body regions colored in grey. (**A**) Vertex to toe; (**B**) Vertex to mid-thigh; (**C**) Skull base to mid-thigh; (**D**) Chest/abdomen/pelvis; (**E**) Abdomen/pelvis; and (**F**) Pelvis. Arms were not included in the scan area due to positioning above the head in protocols C – F. Typical proportions divide the body into eight sections the size of the head, with the torso comprising three sections and the legs four sections. Applying this principle, omitting the area below the mid-thigh (**B**) would mean reducing the scan length to five-eighths. A reduction to CAP (**D**) would accordingly mean a shortening to three-eighths of the scan length, with circa an eighth each for the chest, abdomen, and pelvis. Scan length and scan time are often equivalent

Due to the anatomical overlap, the caudal end of the thorax and the cranial end of the abdomen intersect each other intentionally. Following, hypothetical scan protocols were defined, which were oriented to extensions known in PET/CT imaging or equivalent to anatomical regions known from radiological imaging like CT in subsequent reduction of longitudinal length: Vertex to mid-thigh (PET/CT standard); Skull base to mid-thigh (PET/CT standard); Chest/abdomen/pelvis (CT standard); Abdomen/pelvis (CT standard); and Pelvis (CT standard) (Fig. 1). As the positioning of the arms typically varies between different scan protocols, they were included in the analysis for the vertex-to-toe and the vertex-to-mid-thigh protocols, but not for the other examination protocols, in which the arms are typically positioned above the head.

PSMA-positive tumor lesions were allocated and counted within every region. To assess the relevance, tumor lesions were additionally classified in affected organ systems: local recurrence in prostate bed, lymph node metastases, bone metastases and visceral metastases. Lesions were assigned to the region that covers > 50% of the lesion. Furthermore, the tumor lesion with the smallest and biggest distance from the prostate bed were assessed, as well as the most distant tumor lesion that showed the involvement of a new organ system (local recurrence, lymph nodes, bone, visceral metastases) for the first time.

### Statistics

Data evaluation was performed mostly descriptive. Correlations were calculated using Spearman’s Rho (ρ) Rank Correlation. To determine the PSA limit values, the range means of log(PSA) were determined, i.e., the respective means of the individual ranges. All values were then centered using their range means, and the total standard deviation σ was calculated from these. Finally, the 1.96-fold standard deviation was subtracted from the non-centered range means to determine the lower threshold values of the respective ranges.

## Results

The 200 men included in the study had a mean age of 69.7 years and a median PSA level of 1.1 ng/mL at the time point of the PET/CT. Initial tumor therapy was more frequent surgical prostatectomy and systemic androgen deprivation therapy was performed in a minority of patients (Table 2). PSA did not differ between patients after resection (median 1.1, range 0.03–105.3) and patients after radiation (median 1.1, range 0.1–44.2). Gleason score was most commonly at 7 (45.5%), followed by 8 (19%), 9 (18.5%), 6 (16%), and 10 (1%). Three different PSMA ligands were used, most frequently PSMA-HBED/-11 (82%), followed by PSMA-617 in 14.5% and PSMA-I&T in 3.5% of patients. Mean administered activity was 251.8 (± 11.7) MBq, mean tracer uptake time was 77 (± 15.9 min). The true whole-body scan from vertex to toe needed between 13 and 15 adjoining bed positions.

**Table 2:** Patients’ characteristics

**Age**	Mean	69.7
	SD	7
	Range	46–90
**Primary treatment**	Resection	173/200 (86.5%)
	Radiation	27/200 (13.5%)
**PSA level [ng/ml]**	Mean	3.3
	SD	9
	Median	1.1
	Range	0.03–105.3
**ADT**	Performed priorily	69/200 (34.5%)
	Not performed priorily	131/200 (65.5%)

At least one structural correlate of BCR could be identified in 64% (128/200) of patients, defining the detection rate of the examination, which is strongly dependent on PSA serum levels. Detection rates were poor for very low PSA levels ≤ 0.5 ng/ml. In contrast, higher PSA levels > 3 ng/ml showed nearly complete detection (Table 3). The proportions of PET-positive patients were considered to be the same for the PSMA ligands, PSMA-HBED/-11: 103/164 (62.8%), PSMA-617: 9/29 (65.5%), and PSMA-I&T: 6/7 (85.7%), although the latter, at least, is only partially representative due to the small number of patients.

**Table 3:** Dependency of sensitivity from PSA level

PSA level [ng/ml]	Number of patients (*n* = 200)	Number of PET positive patients (*n* = 128)	Detection rate
≤ 0.5	75	27	36%
> 0.5-1	21	14	67%
> 1–2	32	24	75%
> 2–3	31	23	74%
> 3	41	40	98%

**Fig. 2:**
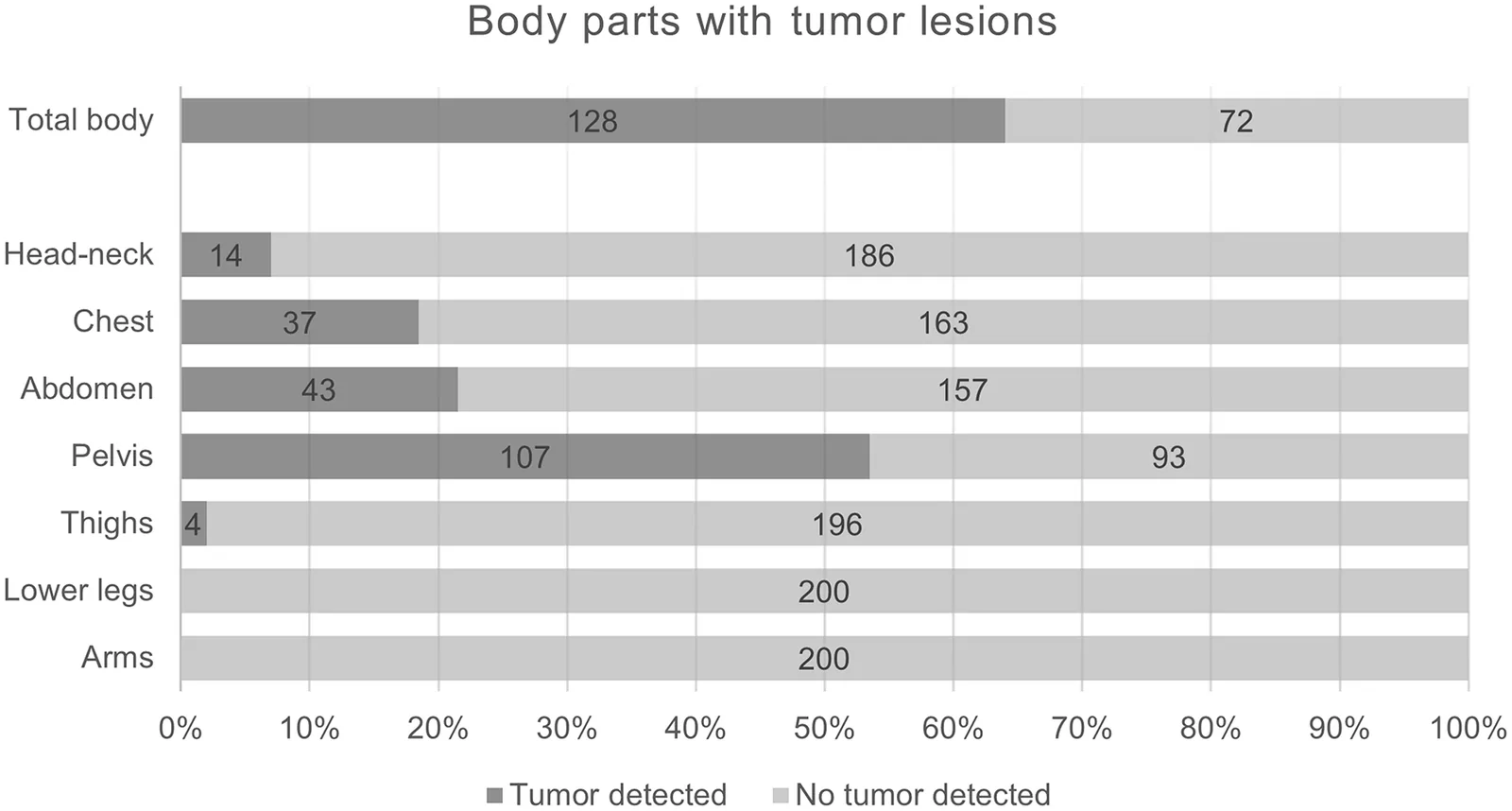
Detected tumor involvement per body part

Evaluation showed local recurrence within the prostate bed in 18.5% (37/200), lymph node metastases in 40% (80/200), osseous metastases in 23.5% (47/200) and visceral metastases in 3.5% (6/200) of patients. Visceral metastases were present in the lung (4 patients) and the liver (1 patient). Averagely 0.9 (171/200) organ systems were detected as tumorous involvement per patient or 1.4 (171/128) organ systems with tumorous involvement per patient with tumor detection in general. In 69 patients (34.5%) the PET/CT showed a tumor manifestation outside the prostate bed or the pelvic lymph nodes. Within separated body regions tumor manifestations were most often detected in the pelvis (53.5%), followed by the abdomen (21.5%), the chest (18.5%), the head-and-neck region (7%), and the thighs (2%), multiple body regions could be involved in the same patient. No tumor manifestation has been detected in the lower legs or in the arms (Fig. 2).

### Protocols with reduced scanning length

#### Vertex to mid-thigh

All possible patients (128/200) would have shown a correlate for the BCR and were therefore still PET-positive. In 2/200 patients (1%) single additional tumor lesions would have been missed compared to true whole-body scans. All tumor-involved organ systems would have been detected correctly.

#### Skull base to mid-thigh

All possible patients (128/200) would have shown a correlate for the BCR and were therefore still PET-positive. In 4/200 patients (2%) single additional tumor lesions would have been missed compared to true whole-body scans. Only in 1/200 patients (0.5%) a metastasis in an otherwise uninvolved organ system would have been missed – i.e. a solitary bone metastasis was localized within the head.

#### Chest/abdomen/pelvis

**Fig. 3:**
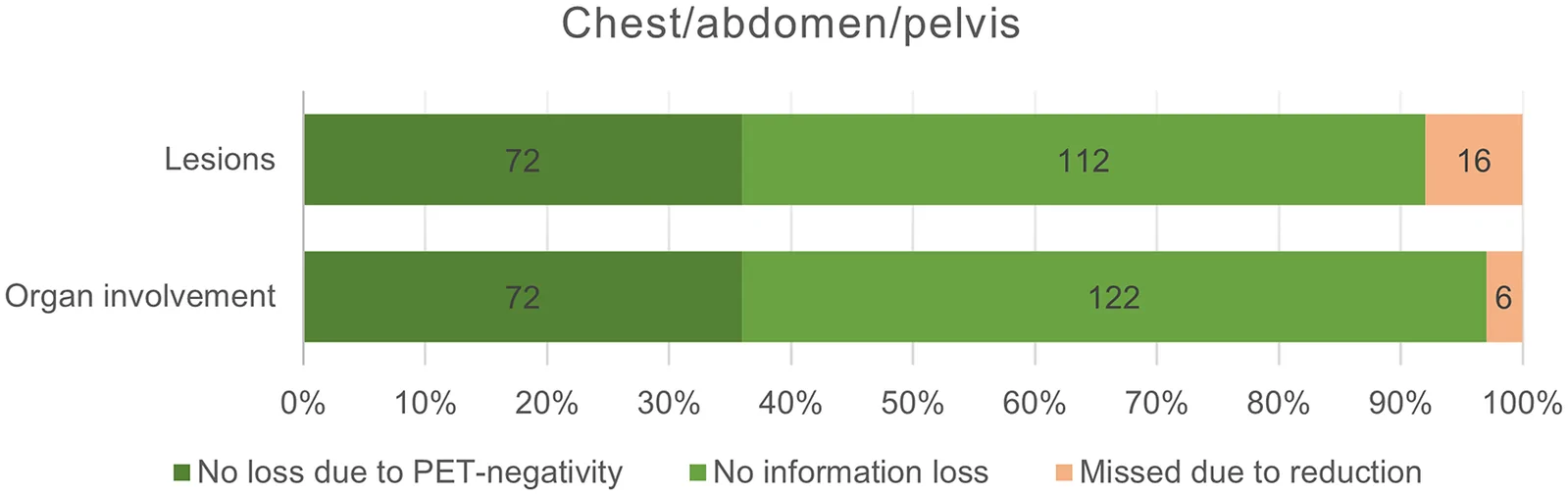
Missed information in a scan of chest/abdomen/pelvis

All possible patients (128/200) would have shown a correlate for the BCR and were therefore still PET-positive. In 16/200 patients (8%) single additional tumor lesions would have been missed, whereas, only in 6/200 patients (3%) metastasized organ systems would have remained undetected as a result of the reduction in the scan area (Fig. 3). When considering only the 128 PET-positive patients, the decrease in the detection rate is 12.5% of patients with respect to all lesions and 5% of patients with respect to the affected organ systems.

#### Abdomen/pelvis

**Fig. 4:**
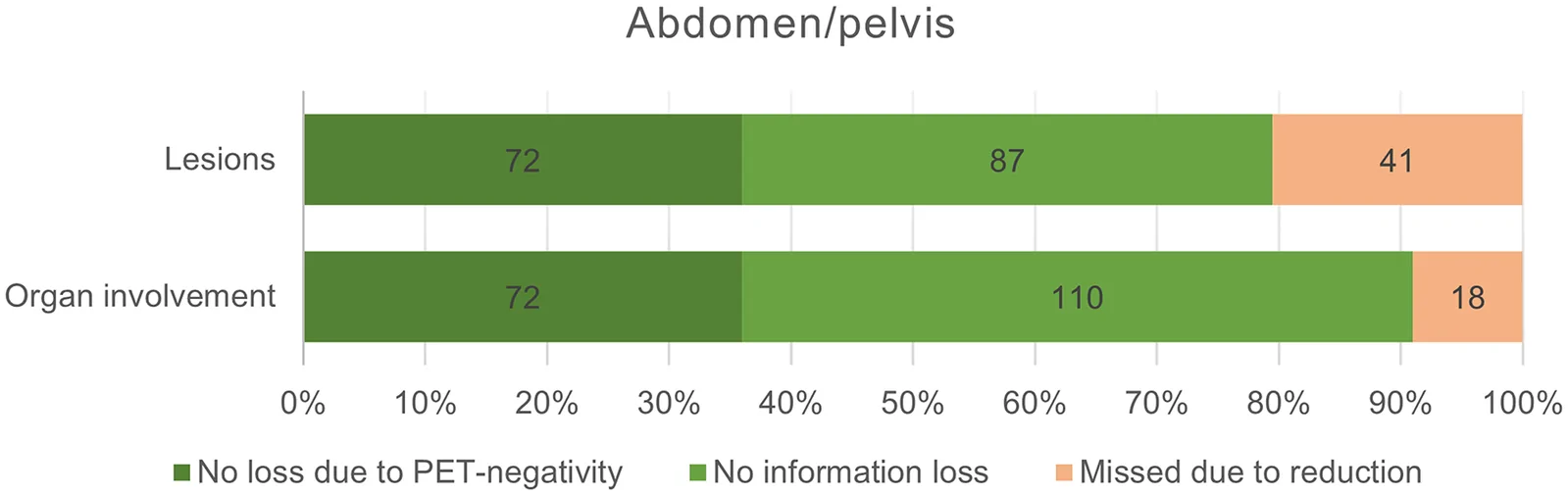
Missed information in a scan of abdomen/pelvis

The detection rate of at least one correlate would have decreased to 122/200 (61%). In 6/200 additional patients (3%) the only correlate(s) would have been missed and the scan became PET-negative. In 41/200 patients (20.5%) single tumor lesions would have been missed, while in 18/200 patients (9%) metastasized organ systems would have remained undetected as a result of the reduction in the scan area (Fig. 4). When considering only the 128 PET-positive patients, the decrease in the detection rate is 32% of patients with respect to all lesions and 14% of patients with respect to the affected organ systems.

#### Pelvis

**Fig. 5:**
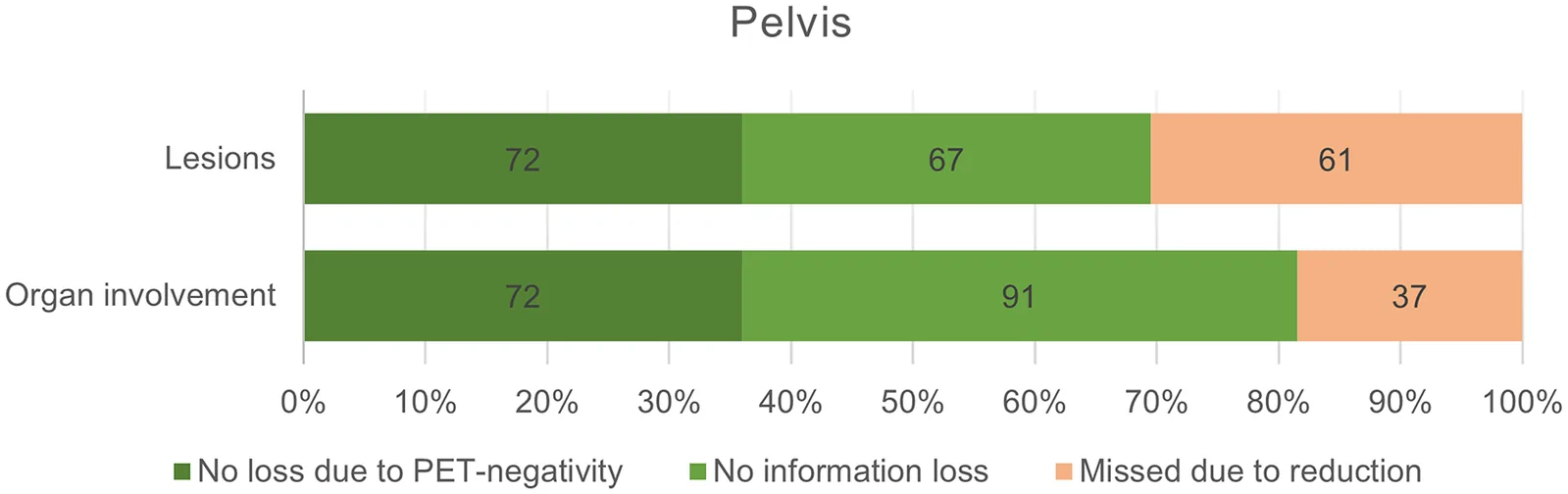
Missed information in a scan of only the pelvis

The detection rate of at least one PET correlate would have decreased to 107/200 patients (53.5%). In 21/200 additional patients (10.5%) the only correlate(s) would have been missed and the scan became PET-negative. In 61/200 patients (30.5%), single tumor lesions would have been missed, while in 37/200 patients (18.5%) actually metastasized organ systems would have remained undetected as a result of the reduction in the scan area (Fig. 5). When considering only the 128 PET-positive patients, the decrease in the detection rate is 48% of patients with respect to all lesions and 29% of patients with respect to the affected organ systems.

### Predictors of extent of metastases

PSA levels showed a strong and significant correlation to the number of metastasis (*ρ* = 0.59, *p* < 0.001). The most distant metastasis was localized cranially from prostate bed in every patient. Distance of farthermost metastasis correlates with PSA levels moderately and significantly (*ρ* = 0.35, *p* < 0.001). Other potential parameters of influence, as age, Gleason score, initial staging, or initial treatment, did not prove sufficient as predictors. With the aim to prospectively identify patients for reduced scanning fields PSA cut-off values were calculated for the respective protocols. A PSA < 0.15 ng/mL was obtained as lower limit to not perform a PSMA-PET/CT. Using this, 12 patients of our cohort would have been omitted to examine, but 3 of them showed a PET correlate of tumor recurrence within the pelvis or abdomen. Using a range of a PSA level between 0.15 and 0.75 ng/mL to assign patients for a PET/CT of only chest/abdomen/pelvis would have let to 77 patients in this protocol of which none missed a single tumor lesion. A level of PSA over 0.75 ng/mL would have let to the recommendation of a whole-body PET/CT for 111 patients (55.5%) of our cohort, of which only 16 (14.4%) particularly needed the whole-body scan to detected all tumor lesions (Table 4). Other differentiations in protocols of abdomen/pelvis and pelvis only or with regard to detection of metastasized organ systems did not lead to feasible cut-off values or results due to the small number of patients in the respective groups.

**Table 4:** Statistical assignment to scan regions in dependency of PSA level

Hypothetical Recommendation	Omit scan	Chest/abdomen/pelvis	True whole-body (vertex to toe)
PSA level (ng/ml)	< 0.15	0.15–0.75	> 0.75
Number (proportion) of patients assigned	12/200 (6%)	77/200 (38.5%)	111/200 (55.5%)
Number (proportion) of patients thereby under-diagnosed	3/12 (25%)	0/77 (0%)	-
Number (proportion) of scans still longer than required	-	45/77 (58.4%)	95/111 (85.6%)

## Discussion

### Results and comparison to literature

The moderate detection rate of PSMA-PET/CT observed in this study is consistent with previous reports in patients with BCR [11–13]. Reduced detection rate was primarily observed in patients with very low PSA levels. PSA values correlated not only with lesion detectability but also with metastatic extent. The distribution of metastases was comparable to published data, with lymph node metastases being most frequent, followed by osseous involvement, while visceral metastases were rare [6, 7]. Metastases in the lower extremities were uncommon, with thigh involvement observed in only a few patients and no metastases detected in the lower legs, in line with prior studies [9]. PSMA-PET/CT enables individualized, metastasis-directed treatment strategies in patients with BCR after initially curative therapy [14]. In our cohort, 34.5% of patients demonstrated metastases beyond the prostate bed and pelvic lymph nodes, potentially avoiding insufficient standard salvage therapy. Conversely, patients without PET-detectable lesions (36%) or with disease confined to the prostate bed and pelvic lymph nodes (29.5%) remained suitable for standard salvage approaches. A negative PSMA-PET/CT scan has been suggested as a predictor of favorable outcome after standard salvage therapy [15]. Independent of disease localization, metastasis-directed treatment strategies for uni- or oligometastatic disease are gaining importance, underscoring the need for accurate imaging of tumor distribution, for which PSMA-PET/CT currently represents the most sensitive modality [16].

The optimal longitudinal scan extent in PET/CT remains a subject of debate. As radiotracers distribute systemically and oncologic PET/CT is primarily used for staging, imaging of the total body is often considered. However, protocols excluding the lower extremities are attractive due to reduced scan time and radiation exposure. Previous studies of ^18^F- fluorodeoxyglucose (FDG) PET/CT across various tumor entities, such as melanoma, sarcoma, breast cancer and lymphoma in children, have demonstrated that lesions in the lower legs are rare and only infrequently affect treatment decisions [17–21]. Consequently, routine whole-body PET/CT from vertex to toe has not been recommended when the primary tumor is not located in the distal extremities [8, 22–26]. Evidence regarding true whole-body PSMA-PET/CT in prostate cancer is limited. Existing studies, in line with our results, reported very low rates of metastases in the lower extremities, almost always occurring in the context of widespread disease and without therapeutic impact [9, 27, 28]. In patients with BCR, lesions in the lower legs were identified in approximately 2–3% of cases, with no exclusive metastases. These findings support the recommendation to omit routine imaging of the lower legs in PSMA-PET/CT for BCR [9].

**Fig. 6:**
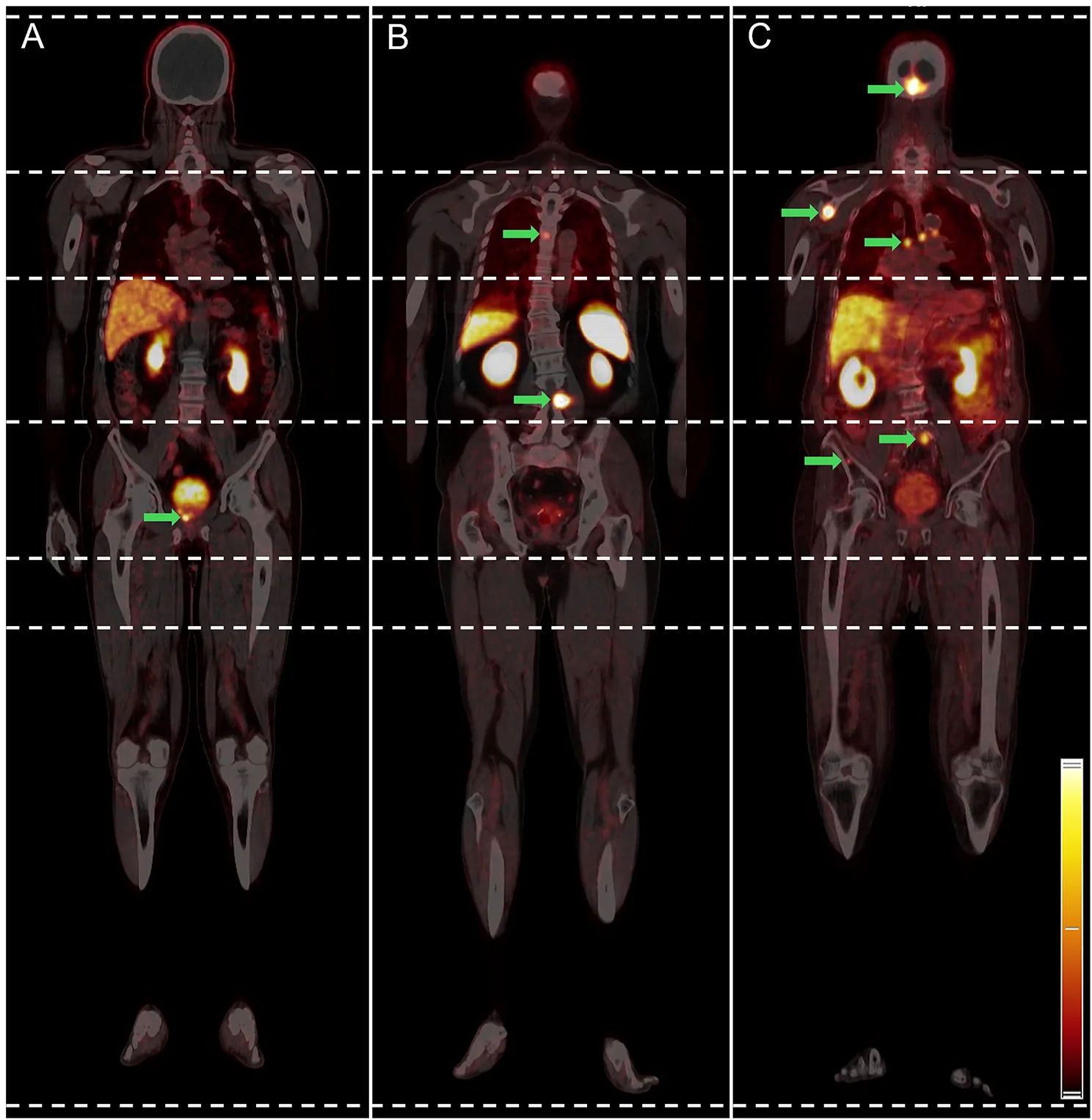
Examples with typical constellations of tumor manifestations in three different patients, considering the scan areas. Dashed lines indicate the limits of vertex, chest, abdomen, pelvis, mid-thigh and toes, while green arrows point to PSMA-positive tumor lesions. **A** (PSA 1.2 ng/ml) shows a solitary local recurrence within the prostate bed, total depiction of the tumor extent would have been able with a scan of only the pelvis. **B** (PSA 1.8 ng/ml) shows two osseous metastases in the lumbar and thoracic spine, total depiction of the tumor extent in the CAP region. All affected organs (bone) would have been detected by the AP region already. **C** (PSA 6.1 ng/ml) shows only a fraction of the patients’ metastases (*n* = 13, osseous metastases in the right ilium, the right scapula and the occipital skull, lymph node metastases are located along the iliac vessels and in the mediastinum, a scan from vertex to mid-thigh was necessary to detect all tumor manifestations. All affected organs (bone, lymph nodes) would have been detected by a scan of the pelvis already

To our knowledge, further reduction of scan length, especially regarding typical sections known from CT or MRI, has not previously been investigated for PSMA-PET/CT in prostate cancer. Prior segmental PET/CT protocols mainly addressed specific clinical questions, such as characterization of pulmonary nodules or assessment of localized therapy response in FDG-PET/CT, rather than staging [29–33]. As this represents a novel approach, we assessed the information loss associated with partial-body imaging protocols and their potential therapeutic impact. Specifically, we analyzed the probability of missing the only metastasis, any metastasis, or a metastasis indicating involvement of a new organ system (Fig. 6). While CAP scans did not miss the only metastasis and missed few lesions overall, AP or pelvis-only protocols resulted in missed solitary metastases and a substantial proportion of total lesions. Because the detection of a newly involved organ system can alter treatment decisions – for example, the first bone metastasis in a patient previously presenting only with lymph node metastases – we also evaluated this aspect. Newly metastasized organ systems would have been overlooked in a relevant proportion of patients, particularly with more restricted protocols.

Different approaches have been used to predict the extent of metastases or suitability for segmental examination protocols before [34, 35]. Among evaluated parameters, PSA level was the only reliable predictor of metastatic burden and distribution. PSA is readily available prior to imaging and thus suitable for prospective protocol adaptation. A PSA threshold of 0.15 ng/mL for omitting PSMA-PET/CT appears unreliable, as a relevant proportion of patients would have harbored detectable disease. By contrast, using a threshold of ≤ 0.75 ng/mL to select patients for a reduced CAP protocol appeared feasible, as no metastases would have been missed in this subgroup. Patients with PSA levels > 0.75 ng/mL might be assigned to whole-body imaging, although this would be necessary in only a minority of cases (14.4%). Overall, nearly half of the cohort presented here (44.5%) could have undergone a reduced FOV scan without loss of diagnostic information if this rule had been applied. The proposed cut-off values reported here were used to formulate hypotheses, as they were calculated and tested using the same 200 patients. Validation in a different cohort would be necessary.

### Clinical implications

Optimization of PSMA-PET/CT protocols offers several advantages, including reduced scan time, lower radiation exposure, and decreased costs. However, it must be acknowledged that the reduction in radiation exposure achieved by avoiding low-dose CT scans of the legs is fairly minimal. Shortened protocols may also allow reduced radiotracer activity if compensated by longer acquisition times per bed position [31]. Given the increasing demand for PSMA-PET/CT in diagnostic and theranostic settings and limited scanner availability, protocol optimization is clinically relevant. Alternatives such as PSMA-scintigraphy are being used so far, for which shortening the scan protocol by excluding the lower legs is being considered feasible as well [36]. Another approach of rationalizing the examination time of true whole-body PET/CT is to selectively reduce the scan time for the legs [37]. Under reasonable assumptions, protocol adaptation could allow a substantial increase in patient throughput, thereby improving access to PSMA-PET/CT for patients with evidence-based indications. Using the rule specified here, that approximately half of PSMA PET/CT scans can be performed either from vertex to mid-thigh or only of the CAP (see Fig. 1 and body proportions; accordingly, vertex to mid-thigh is approximately 5/8, and CAP is approximately 3/8 of the total body length), a halving of the total scan time or performing twice as many examinations compared to true whole-body scans would be possible.

### Limitations and methodology

This study comprises a retrospective analysis of clinical patient data. Since the definition of biochemical recurrence or PSA elevation was based on referral for examination, discrepancies emerged with regard to PSA levels—some of which were very low prior to PET/CT—that did not meet the formal definition of biochemical recurrence. In this regard, the test results for this subgroup of patients may also be true-negatives, if no tumor lesion was detected. The reduction of the scan area is hypothetical, which creates the possibility of evaluating lesions that would have been unrecorded. There is no histological verification of the PET/CT findings evaluated as tumor manifestations. The therapeutic consequences of detected or undetected metastases cannot be comprehensively assessed due to being hypothetical and individualized treatment decisions. To estimate clinical relevance, lesions defining tumor involvement of a new organ system were documented in addition to lesion counts. An external validation of the hypothesis-generating cut-offs is missing. No relevant influence on detection rates was expected from the use of different PSMA ligands [11]. While long-axial FOV PET/CT systems allow single-step whole-body imaging with advantages regarding sensitivity, radiation exposure, and dynamic imaging and have been recommended for PSMA-PET/CT in biochemical recurrence (BCR), their availability remains limited [38–40]. Conventional PET/CT systems with step-and-shoot or continuous-motion acquisition therefore continue to represent the standard of care. Moreover, even most long-axial FOV scanners cannot cover the entire body from vertex to toe in a single acquisition, necessitating some restriction of scan extent.

## Conclusion

PSMA-PET/CT is the most important tool in imaging and treatment-guidance of BCR-PCa. Due to restricted availability, an optimization of scanning protocols is necessary to make this method accessible for every patient fulfilling its evidence-based indication. Based on the results of this study we do not recommend true whole-body scans for patients without specific reasons. A standard scan from vertex to mid-thigh seems sufficient without missing relevant metastases. In cases with low PSA levels, i.e. ≤ 0.75 ng/mL, an even more focused protocol of only chest/abdomen/pelvis can be considered. Segmental approaches limited to the pelvis or abdomen/pelvis only do not meet the objective of a comprehensive recurrence staging.

## Data Availability

The data that support the findings of this study are available from the corresponding author, upon reasonable request.

## Abbreviations

AP: Abdomen/pelvis
BCR: Biochemical recurrence
CAP: Chest/abdomen/pelvis
CT: Computed tomography
FDG: Fluorodeoxyglucose
FOV: Field of view
i.v.: Intravenous
MBq: Megabecquerel
MRI: Magnetic resonance imaging
PCa: Prostate cancer
PET/CT: Positron emission tomography/computed tomography
PSA: Prostate-specific antigen
PSMA: Prostate-specific membrane antigen
